# A Rare Case of Esophageal Dysphagia in Children: Aberrant Right Subclavian Artery

**DOI:** 10.1155/2016/2539374

**Published:** 2016-01-20

**Authors:** Claudia Barone, Nicolina Stefania Carucci, Claudio Romano

**Affiliations:** Pediatric Department, University of Messina, Italy

## Abstract

Dysphagia is an impairment of swallowing that may involve any structures from the mouth to the stomach. Esophageal dysphagia presents with the sensation of food sticking, pain with swallowing, substernal pressure, or chronic heartburn. There are many causes of esophageal dysphagia, such as motility disorders and mechanical and inflammatory diseases. Infrequently dysphagia arises from extrinsic compression of the esophagus from any vascular anomaly of the aortic arch. The most common embryologic abnormality of the aortic arch is aberrant right subclavian artery, clinically known as* arteria lusoria*. This abnormality is usually silent. Here, we report a case of six-year-old child presenting to us with a history of progressive dysphagia without respiratory symptoms. A barium esophagogram showed an increase of the physiological esophageal narrowing at the level of aortic arch, while at esophagogastroduodenoscopy there was an extrinsic pulsatile compression of the posterior portion of the esophagus suggesting an extrinsic compression by an aberrant vessel. Angio-CT (computed tomography) scan confirmed the presence of an aberrant right subclavian artery.

## 1. Introduction 

Swallowing is an important function in maintaining optimal nutritional status [1]. It occurs in three phases: oral, pharyngeal, and esophageal. Dysphagia is any disturbance in swallowing, often described by the patients as a “perception” that there is an impediment to the normal passage of the swallowed material [2]. Oropharyngeal dysphagia involves the initial two phases and is rather common in patients with neurological impairment; esophageal dysphagia occurs at the third phase and is most commonly due to functional causes or anatomic abnormalities affecting the esophagus [3, 4]. Early detection of dysphagia in infants and children is important to prevent or minimize complications because if not diagnosed this medical condition can lead to failure to thrive, aspiration pneumonia, gastroesophageal reflux, and/or the inability to establish and maintain proper nutrition and hydration [5]. A variety of medical conditions cause swallowing disorders in pediatric patients [6]. Aberrant right subclavian artery (ARSA) is a rare cause of dysphagia, but it must be taken into account in the differential diagnosis [7]. The majority of patients with this abnormality remain asymptomatic. In other cases ARSA may cause respiratory symptoms in children and dysphagia, cough, and chest pain in adults [7–10]. We report a rare case of six-year-old child presenting to us with a history of progressive dysphagia.

## 2. Case Report

**Figure 1:**
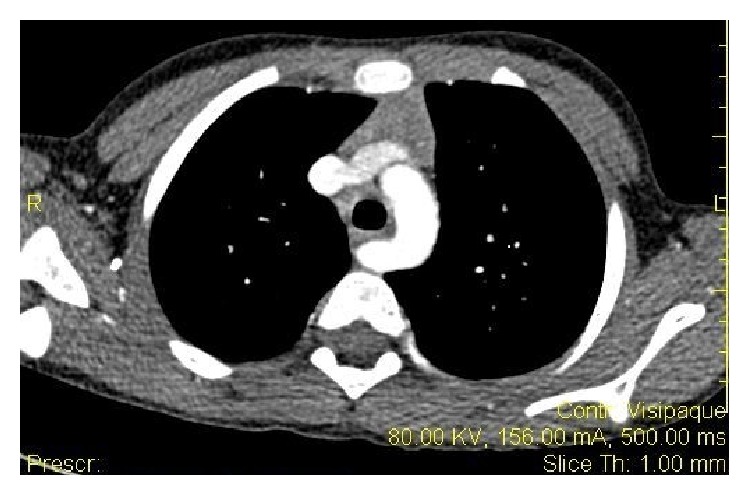
*Angio-computed tomography*: demonstrating the aberrant right subclavian artery compressing the esophagus.

A six-year-old child presented to our hospital with a progressive history of dysphagia, chest pain, and slow feeding. He did not have respiratory symptoms. His examination was normal with weight and length at the 90th percentile for age. His blood tests were normal. There were no abnormalities on ECG or echocardiography. A barium esophagogram showed an increase of the physiological esophageal narrowing at the level of aortic arch, being suggestive of extrinsic compression. At esophagogastroduodenoscopy, the esophageal mucosa was normal in appearance; biopsy examinations confirmed normal mucosa. About 15 cm from the buccal rhyme there was an extrinsic compression of the posterior portion of the esophagus that was pulsatile, suggestive of an aberrant vessel. Angio-CT (computed tomography) scan confirmed an aberrant right subclavian artery compressing the posterior middle third of the thoracic esophagus (Figure 1). This artery originated from an aneurismal dilatation (11 mm), Kommerell's diverticulum. An additional finding was the common origin of the right and left carotid arteries from the aortic arch. The patient's discomfort indicated operative repair of this condition. A vascular clamp was applied to the right subclavian artery at its origin from the aortic arch. This artery was divided and the distal portion was trimmed; then an end-to-side anastomosis was made with the right common carotid artery. Our patient reported complete resolution of his symptoms and tolerated a regular diet without dysphagia.

## 3. Discussion

Dysphagia is defined as difficulty in swallowing food (semisolid or solid), liquid, or both [11]. During normal swallowing, the bolus is propelled from the oral cavity through the pharynx and down the esophagus. Dysphagia occurs when there is a problem with bolus containment and/or propulsion and may occur at the oral, pharyngeal, and/or esophageal phases of swallowing [5, 11–13]. Esophageal dysphagia can arise from a variety of causes such as motility disorders and mechanical and inflammatory diseases [14] shown below.

Causes of esophageal dysphagia in children are as follows

 GERD.
 Eosinophilic esophagitis.
 Tracheoesophageal fistula and esophageal atresia.
 Ingestional injuries:
   Caustic.
   Foreign body.
 Congenital diaphragmatic hernia.
 Cicatricial stenosis.
 Esophageal diverticulae.
 Motor disorders:
   Achalasia.
   Diffuse esophageal spasm.
   Nutcracker esophagus.
   Hypertensive lower esophageal sphincter.
 Extraesophageal compression:
   Mediastinal masses (lymphoma, lymph nodes, and thyromegaly).
   Vascular compression (dysphagia lusoria, dysphagia aortica, and cardiomegaly).
 Systemic diseases:
   Crohn's disease.
   Leiomyomatosis.
 Iatrogenic complications:
   Radiotherapy.
   Drugs.
   Postsurgical complications.

It is rarely caused by extrinsic compression of the esophagus from any vascular anomaly of the aortic arch [15]. ARSA is the most common congenital anomaly of the aortic arch and has a prevalence ranging from 0.5% to 1.8% in the general population [7, 16, 17]. Although most cases of this anomaly are asymptomatic, symptoms may appear when a “ring” completely encircles the trachea or the esophagus. Extrinsic compression of the esophagus may lead to dysphagia [18, 19]. Symptoms, when present, occur at the two extremes of life. In infants, the trachea is compressible; therefore, the typical signs and symptoms of the compression by arteria lusoria are respiratory, such as wheezing, stridor, recurrent pneumonia, and cyanosis. Dysphagia mostly occurs in adults, in whom respiratory symptoms are rare [8, 20]; adequate management of dysphagia includes a detailed history, evaluation with barium radiography, upper endoscopy, and manometry [14]. The best initial diagnosis would be a barium swallow that will allow for visualization of the esophagus via contrast radiography to determine if there is any evidence of a narrowing due to a stricture, an intraluminal mass, or extraluminal compression [4]. In ARSA, barium esophagogram is often suggestive, showing oblique compression of the esophagus at the level of the third and the fourth thoracic vertebrae [16, 21]. Upper gastrointestinal endoscopy may show prominent aortic pulsation but it is not necessary for the diagnosis. CT or MRI (magnetic resonance imaging) angiography has replaced conventional angiography and is considered the gold standard for the diagnosis. It not only does confirm the diagnosis but also helps to plan the operation and to exclude aneurysm of the aorta or presence of other associated anomalies [17]. Echocardiography has the advantage of a comprehensive assessment of intracardiac anatomy and function. In the presence of respiratory symptoms, the evaluation normally begins with chest radiography [22]. When noisy breathing, stridor, or brassy cough is evident flexible airway endoscopy is the procedure of choice. Despite the accuracy of both MRI and CT in evaluating the nature of the vascular compression of the airways, current techniques do not reliably distinguish between dynamic and static airway narrowing, and coexisting (laryngo) tracheo- or bronchomalacia or tracheal rings can be differentiated with flexible bronchoscopy [23]. Manometry cannot be used to diagnose the condition nor has it been of any assistance in distinguishing which patients may benefit from surgery [21]. The treatment depends on the symptoms, age comorbidity, and concomitant vascular abnormalities of each patient [7]. Surgical approach is indicated when ARSA is symptomatic or has evidence of aneurysm [9]. Various surgical approaches can be used, each with its own advantages and limitations [24]. A symptomatic ARSA can be safely repaired through minimally invasive surgery and endovascular techniques, although symptoms do not always regress. Aggressive treatment of an aneurysmal lusorian artery should be proposed, given the rapid natural evolution towards rupture and high mortality of this complication, despite high operative mortality associated with this elective procedure. Endovascular exclusion is an option in patients who are not good surgical candidates [19].

Summarizing, dysphagia is any disturbance in swallowing that may involve oropharyngeal or esophageal phase. Esophageal dysphagia can arise from a variety of disorders and it is rarely caused by arteria lusoria. Symptoms of ARSA are usually different according to age: children predominantly have respiratory symptoms while dysphagia, cough, and chest pain occur mostly in adults. On the contrary, our patient presented with a long history of dysphagia, associated with chest pain and slow feeding. In conclusion, ARSA is a rare cause of dysphagia but it should be taken into account in the differential diagnosis. It is important to be aware of its existence and features to allow an early diagnosis and avoid unnecessary therapeutic interventions. Only an early detection can prevent or minimize complications: unlike adults, children have rapidly developing body systems and even short-term dysphagia can have a detrimental effect on dietary intake. As a result, swallowing difficulties can interrupt physical growth and cognitive development and cause serious long-term sequelae. For all these reasons, it is imperative to accurately identify and appropriately manage dysphagia in pediatric population.

## Abbreviation

ARSA: Aberrant right subclavian artery.
